# Endocrine-Disrupting Chemicals and Male Infertility: Mechanisms, Risks, and Regulatory Challenges

**DOI:** 10.3390/jox15050165

**Published:** 2025-10-13

**Authors:** Sofoklis Stavros, Nikolaos Kathopoulis, Efthalia Moustakli, Anastasios Potiris, Ismini Anagnostaki, Spyridon Topis, Nefeli Arkouli, Konstantinos Louis, Charalampos Theofanakis, Themos Grigoriadis, Nikolaos Thomakos, Athanasios Zikopoulos

**Affiliations:** 1 Third Department of Obstetrics and Gynecology, University General Hospital “ATTIKON”, Medical School, National and Kapodistrian University of Athens, 12462 Athens, Greece; sfstavrou@med.uoa.gr (S.S.); isanagnostaki3@gmail.com (I.A.); spyros.topis1996@gmail.com (S.T.); kostaslouisss@gmail.com (K.L.); ch.theofanakis@gmail.com (C.T.); thanzik92@gmail.com (A.Z.); 2 First Department of Obstetrics and Gynecology, Alexandra Hospital, Medical School, National and Kapodistrian University of Athens, 11528 Athens, Greece; nickatho@gmail.com (N.K.); tgregos@med.uoa.gr (T.G.); nthomakos@med.uoa.gr (N.T.); 3 Department of Nursing, School of Health Sciences, University of Ioannina, 4th Kilometer National Highway Str. Ioannina-Athens, 45500 Ioannina, Greece; ef.moustakli@uoi.gr; 4 Department of Obstetrics and Gynecology, Tzanio Hospital, 18536 Piraeus, Greece; narkouli@windowslive.com

**Keywords:** spermatogenesis, hormonal imbalance, oxidative stress, epigenetic modifications, environmental exposure, regulatory challenges

## Abstract

Male reproductive health is increasingly threatened by endocrine-disrupting chemicals (EDCs), which interfere with hormonal homeostasis and reproductive physiology. Rising rates of male infertility have been linked to greater exposure to pollutants such as heavy metals, phthalates, pesticides, and bisphenol A. These compounds act through multiple mechanisms, including oxidative stress, apoptosis, receptor-mediated disruption of estrogenic and androgenic signaling, alterations in the hypothalamic–pituitary–gonadal (HPG) axis, and heritable epigenetic changes. Such disruptions impair key outcomes like sperm concentration, motility, morphology, DNA integrity, and steroidogenesis. Evidence from animal studies and human epidemiology consistently demonstrates these harmful effects, with biomarkers of EDC exposure correlating with reduced semen quality, hormonal imbalances, and infertility. Beyond individual health, infertility linked to EDCs carries significant social and economic costs. This review evaluates regulatory frameworks, highlights methodological challenges in risk assessment, and synthesizes mechanistic and clinical evidence. Particular attention is given to unresolved issues such as non-monotonic dose responses, mixture effects, low-dose exposures, and transgenerational impacts. Future priorities include refining biomonitoring, addressing mixture risks, and strengthening international regulation. By integrating mechanistic, clinical, and policy insights, this review underscores the urgent need for strategies to mitigate EDC-related threats to male reproductive health.

## 1. Introduction

Exogenous substances known as endocrine-disrupting chemicals (EDCs) disrupt the regular control of physiological processes by interfering with the synthesis, secretion, transport, binding, action, or clearance of endogenous hormones. Today’s world is full of these compounds, which originate from heavy metals, plastics, industrial products, pesticides, pharmaceuticals, and personal care items [1,2,3]. Since EDCs can be inhaled through contaminated air, consumed through contaminated food and water, or come into contact with consumer products through the skin, they are a common public health concern [4].

Infertility rates have risen, semen quality has changed, and sperm counts have been steadily declining over the past 50 years, raising concerns about male reproductive health [5]. A groundbreaking meta-analysis that revealed an approximately 50% drop in sperm concentration in males from industrialized regions between 1973 and 2011 triggered debates about environmental concerns, such as endocrine disruptors [6]. Most recent meta-analyses confirm that declining trends have continued into the 21st century, underscoring persistent global concerns. Infertility has been designated as a public health priority by the World Health Organization (WHO), as male factors account for over half of all cases. Understanding the role of EDCs from a biological and policy perspective is therefore more crucial than ever [7].

In recent decades, increasing research has connected exposure to EDC to declining male reproductive health [8]. Epidemiological studies have connected EDC indicators to lower semen quality, hypospadias, cryptorchidism, hormonal abnormalities, and an elevated risk of infertility. Although this association is still up for debate, other research has also suggested connections to testicular cancer. Experimental studies in vitro and in animal models have also shed light on the mechanistic basis of these reproductive effects by revealing the molecular targets and pathways affected by EDCs [7,9,10].

EDCs can interfere with male reproductive health through various processes, including receptor binding, disruption of the hypothalamic–pituitary–gonadal (HPG) axis, development of oxidative stress, apoptosis in testicular cells, and epigenetic modifications. These mechanisms and their implications will be discussed in detail in Section 2.1, Section 2.2, Section 2.3 and Section 2.4 [11,12,13].

The male reproductive system is particularly susceptible to EDCs because spermatogenesis and steroidogenesis are tightly regulated processes that rely on accurate hormonal communication [7]. Disruption of these pathways can lead to lower sperm concentration, motility, and shape, altered testosterone production, and decreased DNA integrity, all of which can compromise fertility [14]. Despite gains in knowledge, the cumulative effects of numerous EDCs, critical windows of exposure, dose–response relationships, and long-term reproductive effects in humans are still unknown [15,16]. Uncertainties also stem from non-monotonic dose–response relationships and mixture effects, two particularly debated aspects of EDC toxicology.

This review aims to provide a comprehensive overview of the current state of knowledge about the biological mechanisms by which EDCs affect male fertility. We highlight reproductive results, endocrine disruption mechanisms, and new study areas by combining data from molecular, cellular, animal, and human investigations. To reduce the reproductive risks associated with exposure to EDCs, evidence-based solutions must be designed, and public health policy and regulatory frameworks must be guided by an understanding of these pathways.

Furthermore, this review highlights how urgent it is to advance regulation and research. Evidence from basic research, epidemiology, and risk assessment must be harmonized as global organizations like the WHO, the U.S. Environmental Protection Agency (EPA), and the European Food Safety Authority (EFSA) demand more thorough testing of endocrine-active compounds. Unlike previous reviews, our synthesis integrates mechanistic, clinical, and regulatory perspectives, highlighting the translational relevance of mechanistic evidence for risk assessment.

## 2. Mechanisms of Endocrine Disruption

Multiple interrelated pathways are utilized by EDCs to impact spermatogenesis, male fertility, and hormonal regulation. Both experimental interpretation and regulatory evaluation are made more difficult by these processes, which operate at the molecular, cellular, and systemic levels and frequently in a dose- and timing-dependent manner. The main biological pathways affected by EDCs [17] are summarized in Table 1 and discussed in detail in the following subsections.

### 2.1. Hormone Receptor Interaction

EDCs can change receptor-mediated signaling pathways by imitating or opposing natural hormones [18]. While certain EDCs interfere with androgen receptors (AR) or thyroid hormone receptors, many EDCs function as agonists or antagonists of estrogen receptors (ER), but others also disrupt androgen (AR) and thyroid hormone receptors, highlighting the broad spectrum of receptor families affected. For example, ERα and ERβ are bound by the common plasticizer bisphenol A (BPA), which causes activation of estrogen-responsive genes in tissues where this is normally absent or dysregulated [1,19]. Quantitative studies report that BPA exhibits nanomolar binding affinities (Ki ≈ 5–10 nM) for ERα/ERβ, leading to upregulation of estrogen-responsive transcription in Sertoli cells. Similarly, PCBs disrupt receptors across chemical classes by interfering with thyroid signaling, while vinclozolin functions as a strong AR antagonist with IC50 values below 1 μM. By inhibiting steroidogenic enzymes, phthalates—which are frequently present in plastics and personal hygiene products—decrease testosterone production, with animal studies showing up to 40% reductions in serum testosterone and significant impairments in spermatogenesis. EDCs interfere directly with hormone receptors, upsetting the delicate equilibrium of endocrine communication that is necessary for male reproduction [9,20].

Some EDCs work through non-genomic pathways in addition to traditional receptor binding, immediately triggering calcium signaling and kinase cascades. For example, BPA can rapidly activate MAPK/ERK signaling and induce Ca^2+^ influx in Sertoli cells, disrupting cell communication critical for germ cell support. Predicting biological consequences is made more difficult by these effects, which show that receptor interactions can affect larger signaling networks in addition to transcriptional regulation [21]. The systemic character of endocrine disturbance is highlighted by receptor cross-talk with thyroid and metabolic pathways, which connects metabolic and developmental problems with reproductive damage. Large cohort studies have shown that males in the highest quartile of urinary phthalate metabolites have significantly decreased sperm motility and, on average, 12–15% lower serum testosterone levels than those in low-exposure groups. These mechanisms are supported by human epidemiological evidence. Therefore, these receptor-mediated changes represent a crucial first step that connects environmental exposure to poor male reproductive outcomes.

### 2.2. HPG Axis Interference

The regulation of spermatogenesis and steroidogenesis depends on the HPG axis. EDCs. EDCs can change hypothalamic gonadotropin-releasing hormone (GnRH) secretion and function, as well as the pituitary gland’s follicle-stimulating hormone (FSH) and luteinizing hormone (LH) production [19]. Disruption of this hormonal axis affects Leydig and Sertoli cell activity and leads to reduced testosterone production, poor sperm maturation, and altered seminiferous tubule architecture. EDC exposure is linked to altered LH/FSH ratios and decreased serum testosterone, according to both experimental and clinical research. According to epidemiological research, males who have urinary phthalate metabolite concentrations in the highest quartile have dramatically altered LH/FSH ratios and serum testosterone levels that are about 12% lower than those of low-exposure groups. In cross-sectional cohorts, BPA has been linked to delayed sperm maturation and a 10–15% decrease in blood testosterone levels. Similar results have been found for this compound. This data emphasizes how important a function disruption of the HPG axis is to male reproduction [20,21,22].

Recent studies suggest that early childhood exposure or perinatal exposure may induce long-lasting programming effects on the HPG axis that persist into adulthood. According to longitudinal cohorts, exposure to phthalates or organophosphate pesticides during pregnancy is linked to changed sex steroid profiles during adolescence and a delayed pubertal onset by 6–12 months [22,23]. In addition to reducing fertility, such disturbances may increase susceptibility to endocrine-related diseases later in life. Cross-species research has validated the evolutionary conservation of these mechanisms; after low-dose EDC exposure, rodent models exhibit decreased GnRH neuron activity and disturbed pulsatility of LH secretion [24]. EDC exposure can downregulate hypothalamic Kiss1 expression, which further impairs HPG axis function. Recent research further emphasizes kisspeptin signaling as a critical upstream regulator of GnRH release Clinical observations of altered LH/FSH ratios and lower testosterone, and delayed puberty in males with higher urinary EDC biomarkers are thus in line with experimental evidence showing that disruption of GnRH, LH, and FSH signaling pathways is a significant target of endocrine disruption.

### 2.3. Epigenetic Modifications

Histone modifications, DNA methylation, and non-coding RNA regulation are among the epigenetic alterations that EDCs can cause. Important genes for spermatogenesis and testicular development may have their expression disrupted, including genes regulating meiosis, germ cell differentiation, and steroidogenesis [23,24]. Crucially, some epigenetic changes may last for multiple generations, potentially transmitting increased susceptibility to reproductive dysfunction. Animal studies have shown that exposure to BPA or phthalates during crucial developmental windows such as fetal or perinatal development can alter sperm epigenetic profiles, with transgenerational effects observed up to the F3 generation. For instance, chronic DNA hypomethylation at steroidogenic gene promoter regions and 20–30% decreases in sperm counts in male offspring have been associated with prenatal DEHP exposure in rodents [25,26].

Recent human studies have identified EDC-associated changes in histone retention, microRNA expression, and sperm DNA methylation patterns, confirming that these effects are not limited to animal models. Such alterations may serve as biomarkers of prior exposure and could predict reproductive risk [25]. For example, BPA-exposed men have shown altered sperm DNA methylation at imprinted genes. There are significant ramifications for public health and the danger of intergenerational disease due to the idea of “epigenetic inheritance,” which emphasizes how male reproductive toxicity may affect the health of children [26,27]. The translational importance of this pathway is strengthened by animal research showing EDC-induced epigenetic changes that coincide with new human findings, such as changed sperm DNA methylation profiles in men exposed to BPA.

### 2.4. OS and Apoptosis

By generating reactive oxygen species (ROS) in testicular tissue, many EDCs induce OS. Elevated ROS levels trigger apoptosis in Leydig, Sertoli, and germ cells, while also causing lipid peroxidation, protein oxidation, and DNA strand breaks [7,28]. Comparing men in the highest quartile of BPA or phthalate exposure to controls, meta-analyses show that their seminal ROS levels are 25–30% higher and their sperm DFI is 1.4–1.6 times higher. Infertility is exacerbated by this oxidative damage, which impairs sperm motility, viability, and DNA integrity. Oxidative stress is a major mediator of EDC toxicity, as evidenced by studies that demonstrate antioxidants can partially attenuate EDC-induced testicular injury [29,30].

As mitochondrial dysfunction is now understood to be a key factor in EDC-induced reproductive damage, it exacerbates oxidative imbalance [31,32]. The production of ATP, which is necessary for motility, is compromised by the malfunctioning of sperm mitochondria, which are particularly susceptible to oxidative damage [33]. A vicious cycle in which oxidative damage leads to programmed cell death, further reducing spermatogenic capacity, is highlighted by the interaction between ROS and apoptosis. Although there is currently little clinical evidence though human studies have correlated high seminal ROS with increased DNA fragmentation index (DFI) and reduced motility. This route offers a viable target for therapeutic intervention, such as antioxidant supplementation [34]. Agents including vitamin E, coenzyme Q10, N-acetylcysteine, and chlorogenic acid have shown protective effects in experimental and clinical settings. Clinical observations showing that high seminal ROS concentration correlates with a higher sperm DNA fragmentation index and decreased motility are consistent with oxidative damage observed in experimental settings.

### 2.5. Methodological Challenges

The precision of exposure assessment is a recurring issue in this field. The majority of epidemiological research uses single-spot blood or urine biomarkers, which might not adequately reflect the low-dose, variable, and chronic character of EDC exposure. Many non-persistent compounds have short biological half-lives, which makes interpretation more difficult and repeatability lessened. Moreover, chemical mixtures are rarely taken into consideration by conventional toxicological testing, even though complex “cocktails” are a common occurrence in the real world. Non-monotonic dose–response relationships and these mixing effects continue to be significant barriers to risk characterization. Standardized biomonitoring procedures, sophisticated exposomic tools, and computer models that can incorporate several exposures over time will be necessary to address these challenges.

## 3. Impact on Male Fertility

Male reproductive health has been linked more and more to exposure to EDCs. Fertility is ultimately compromised by the effects, which extend to the molecular, cellular, and physiological levels. Multiple aspects of male reproductive health are susceptible to EDC exposure, as demonstrated through experimental, epidemiological, and clinical research [7,35]. Table 2 provides an overview of the main reproductive effects associated with EDC exposure.

### 3.1. Sperm Quality Parameters

Sperm quality, including morphology, motility, and viability, is a central determinant of male reproductive health since spermatogenesis is extremely sensitive to changes in hormones and the environment. Abnormal morphology and decreased sperm quantity, motility, and viability have been associated with EDCs [36,37]. For instance, exposure to BPA and phthalates has been linked to reduced sperm motility and count in both human and animal models. Similar effects have been reported with organochlorine pesticides and heavy metals such as cadmium and lead. EDC exposure increases DNA fragmentation, induces chromatin condensation defects, and elevates oxidative DNA damage [38,39].

Advanced sperm function assays, which go beyond simple semen analysis, have shown that men exposed to high amounts of EDCs had impaired acrosome reaction, changed mitochondrial activity, and increased DNA fragmentation index (DFI). Such functional abnormalities, even when standard semen measurements are within normal reference ranges, provide mechanistic linkages between exposure and subfertility [7,40].

### 3.2. Hormonal Imbalance

Due to their disruption of endocrine homeostasis, EDCs frequently result in changed ratios of follicle-stimulating hormone (FSH) to luteinizing hormone (LH), decreased testosterone levels, and impaired steroidogenesis. Reduced testosterone contributes to impaired spermatogenesis, decreased libido, and altered development or maintenance of secondary sexual characteristics [41,42]. The clinical significance of EDC-mediated hormonal disruption has been highlighted by human epidemiological investigations that have shown associations between decreased serum testosterone and increased urine phthalate metabolites or BPA levels [35,43].

Additionally, recent research indicates that long-term exposure to low-dose EDC combinations may result in mild but significant changes in the levels of circulating sex steroids [44]. Some demographic cohorts, for instance, have reported altered estradiol: testosterone ratios and decreased inhibin B levels, indicating broader endocrine implications beyond testosterone alone. These results highlight the systemic nature of how the EDC affects reproductive endocrinology [45].

### 3.3. Testicular Morphology and Function

The testes may undergo structural and functional changes as a result of EDC exposure. Degeneration of seminiferous tubules, vacuolization of Sertoli cells, and decreased Leydig cell numbers are all revealed by histopathological investigations [15]. Spermatogenesis and steroid synthesis are directly jeopardized by these structural alterations. Testicular toxicity is dose- and age-dependent in animal studies, highlighting the testes’ susceptibility to EDC exposure during crucial developmental windows [38].

Furthermore, exposure to EDC has been linked to disruption of the blood-testis barrier (BTB), which jeopardizes the immune-privileged milieu necessary for the maturation of germ cells [46,47]. Testicular function has been further compromised by changes in interstitial tissue remodeling and testicular vascularization. The notion that EDCs impair male fertility via modifying testicular architecture in addition to endocrine routes is supported by these structural and microenvironmental alterations [48].

### 3.4. Clinical and Epidemiological Evidence

Male infertility is linked to EDC exposure, according to an increasing amount of epidemiological data. Environmental pollutants like BPA, phthalates, and organochlorine insecticides have been linked in cross-sectional and longitudinal studies to decreased semen quality or infertility [49,50]. The combined results of mechanistic research and animal trials suggest the causal involvement of EDCs in male reproductive failure, even though it is difficult to prove causation in humans due to confounding variables. These results highlight the necessity of ongoing risk assessment, biomonitoring, and preventative measures [1,7].

Interestingly, multicenter cohort studies have consistently shown links between blood or urine biomarkers of EDC exposure and poorer fertility outcomes, such as a longer time to conception and lower assisted reproductive technology (ART) success rates [35,51]. Additional evidence of dose–response correlations between high-level exposure and poor reproductive outcomes comes from occupational cohorts, such as industrial and agricultural workers. When combined, these results support the practical significance of EDC exposure for male reproductive health [52].

## 4. Risk Assessment and Regulatory Perspectives

Comprehensive risk assessment and regulatory oversight are required due to the pervasiveness of EDCs in consumer goods, food systems, and the environment. The impact of EDCs on male reproductive health must be reduced, and this requires an understanding of exposure pathways, dose–response relationships, and susceptible groups [51,53]. Table 3 summarizes the main aspects of exposure routes, biomonitoring strategies, regulatory frameworks, and future challenges.

### 4.1. Exposure Routes

EDCs can enter the human body through a variety of routes. One major source is the oral consumption of contaminated food and water, especially for chemicals like BPA and other pesticide residues. Particularly in indoor and occupational settings, inhaling volatile substances such as flame retardants and some industrial solvents increases overall exposure [4,45]. Furthermore, the absorption of phthalates, parabens, and other EDCs is facilitated by skin contact with cosmetics, personal care products, and plastic-containing goods. When evaluating the hazards to human health, cumulative exposure from these several channels raises the possibility of endocrine disruption and underlines the importance of adopting chemical combinations and long-term low-dose effects into consideration [54,55].

Recent studies suggest that indoor environments—where people spend the majority of their time—may represent significant, underappreciated sources of exposure through dust, microplastics, and volatile compounds [56]. Occupational exposures, such as those seen in the chemical, plastics, and agricultural sectors, are also high-risk situations that highlight the necessity of specific protective measures. Crucially, maternal transfer exposures during pregnancy and the early years of life demonstrate that exposure pathways span generations [57,58].

### 4.2. Biomonitoring and Risk Assessment

Urine, blood, semen, and tissue samples are biological matrices used in biomonitoring studies to measure EDC concentrations and link exposure to reproductive outcomes [59]. Risk assessment includes identifying hazards and doing a mechanistic examination in addition to establishing the reference dosages, acceptable daily intake (ADI), and no-observed-adverse-effect levels (NOAEL). However, low-dose, non-monotonic dose–response curves and combined exposures from chemical combinations cause established risk assessment methods to frequently underestimate the complexity of EDC effects [60,61].

High-resolution mass spectrometry and multi-omics integration are increasingly employed in biomonitoring to simultaneously detect multiple chemicals and identify novel biomarkers of exposure and effect [62]. Coordinated biomonitoring initiatives can inform population-level risk assessment, as exemplified by international consortia such as HBM4EU (Human Biomonitoring for Europe). However, challenges remain in standardizing international protocols and linking biomonitoring data to specific health outcomes [63].

### 4.3. Regulatory Frameworks

Guidelines and laws have been established by a number of regulatory bodies to control the dangers related to EDCs [64,65]. Structured methods for evaluating chemical safety, including reproductive toxicity, are offered by the EFSA guidelines and the REACH (Registration, Evaluation, Authorization, and Restriction of Chemicals) regulation in the European Union [66]. The FDA and the EPA in the US are in charge of evaluating and regulating substances that have the potential to disrupt hormones, especially those found in consumer goods, pesticides, and materials that come into contact with food. Globally, agencies like the United Nations Environment Programme (UNEP) and the WHO provide guidelines for environmental monitoring and risk assessment for public health [4,64]. Significant gaps still exist in spite of these frameworks. The endocrine activity of many chemicals has not been sufficiently investigated, and existing laws frequently fail to take into consideration the effects of chemical combinations, exposure during sensitive developmental windows, or prolonged low-dose exposures [64,67].

Significant variations between jurisdictions are revealed by a comparative study. With stricter REACH regulations and targeted bans on certain phthalates and bisphenols, the EU has adopted a preventive approach. The US regulatory system, however, emphasizes risk–benefit analysis, which often causes delays or partial restrictions [68]. While Asian countries like China and Japan are increasingly adopting EDC regulations, regional harmonization remains limited. This patchwork of standards creates regulatory gaps that allow dangerous compounds to continue circulating globally.

### 4.4. Challenges and Future Directions

Combining toxicological, epidemiological, and mechanistic data is necessary for effective regulation. To forecast EDC activity and improve risk assessment, sophisticated techniques, including computational modeling, omics technology, and in vitro high-throughput screening, are being used more and more [69,70]. Improving regulatory requirements to take into consideration new data on low-dose and transgenerational impacts, as well as minimizing exposure, especially during crucial periods of male reproductive development, should be the top priorities of public health initiatives [52,71].

Addressing the so-called “cocktail effect,” in which several substances with poor individual effects combine to cause notable negative consequences, is another difficulty. Such interactions are often overlooked by current testing procedures [72]. Furthermore, populations are exposed for years or decades before protective measures are implemented due to the delay between scientific discovery and regulatory implementation [73]. Important next measures include enhancing international cooperation, revising testing protocols to incorporate endocrine activity screening, and guaranteeing transparency in chemical safety data.

Effective regulation will ultimately rely on integrating public health, policy, and research. Reducing the reproductive health impact of EDCs could be accelerated by including socioeconomic assessments, environmental justice considerations, and citizen science activities into regulatory decision-making [74].

## 5. Future Directions

Even while our knowledge of how EDCs affect male fertility has advanced significantly, there are still many unanswered questions that need to be addressed [15]. Since humans are rarely exposed to a single chemical, characterizing cumulative exposures to numerous EDCs is an important field. Conventional risk assessment methods do not adequately account for the additive, synergistic, or antagonistic effects that can result from chemical interactions [75].

The identification of sensitive windows of exposure is another crucial area, especially during fetal development, infancy, and adolescence, when spermatogenesis and the HPG axis are particularly vulnerable to disturbance. To evaluate transgenerational effects and correlate early-life exposure with adult reproductive outcomes, longitudinal cohort studies are required [2,76,77]. Considering that male-mediated epigenetic inheritance may greatly influence developmental trajectories, these investigations should also incorporate parental contributions to offspring health.

Novel technologies, such as computational modeling, omics-based methods (genomics, epigenomics, proteomics), and high-throughput in vitro screening, present chances to clarify mechanistic pathways and more accurately forecast chemical toxicity [78]. Biomarkers of exposure, vulnerability, and early reproductive dysfunction can be found with the aid of such integrative techniques [79,80,81]. Artificial intelligence (AI) and multi-omics integration in particular show promise for detecting predictive indicators of susceptibility, allowing for earlier interventions and customized risk assessments [82].

Clinical practice implementation of mechanistic findings is another crucial avenue. In cases of idiopathic infertility, urologists, endocrinologists, and fertility experts are becoming more aware of the part environmental exposures play. Along with routine semen analysis, the development of diagnostic panels that include exposure biomarkers may enhance clinical judgment and provide customized patient care. Furthermore, early exposure and lifestyle changes may be possible if EDC risk assessment is incorporated into preconception counseling [83,84].

Research ought to be conducted on intervention tactics such as dietary changes, antioxidant supplements, and laws meant to limit exposure to recognized EDCs [85]. For example, diets rich in antioxidants and a reduction in the intake of foods packaged in plastic have been associated with improved sperm parameters and reduced internal EDC burdens. Laws pertaining to workplace safety, stricter consumer product standards, and more chemical labeling are important preventive strategies [86].

Finally, international public health education and policy measures are crucial to lowering the reproductive dangers posed by environmental endocrine disruptors [4]. Cross-sector cooperation, global harmonization of regulatory frameworks, and more awareness campaigns would be necessary to reduce exposure disparities among populations. Addressing EDC-related infertility requires coordinated societal efforts that include clinical care, lab research, and public health policy in addition to scientific breakthroughs [35].

## 6. Conclusions

Endocrine-disrupting substances pose a serious and expanding risk to the reproductive health of men. EDCs affect spermatogenesis, steroidogenesis, and sperm quality through a variety of pathways, including hormone receptor interaction, interference with the HPG axis, oxidative stress, apoptosis, and epigenetic alterations. Experimental, epidemiological, and clinical evidence have shown that exposure to pollutants such as bisphenol A, phthalates, pesticides, and heavy metals is associated with reduced sperm concentration, motility, morphology, and DNA integrity in addition to hormonal disruptions.

There are still significant knowledge gaps about low-dose effects, chemical combinations, and transgenerational implications despite advancements in regulatory frameworks and risk assessment methods intended to reduce EDC exposure. Understanding the complex relationships between EDCs and male reproductive health will require future studies that integrate mechanistic, epidemiological, and omics-based methodologies. Specifically, combining molecular biology with advanced computer models and large cohort data will be necessary to detect exposure and illness trends in the real world.

This issue has significant therapeutic, societal, and policy implications that extend beyond advancements in technology. Including an assessment of environmental exposure in the diagnosis of infertility may enable doctors to treat these patients more effectively. Improving bio-monitoring systems, harmonizing international rules, and tackling the financial implications of infertility ought to be top goals for legislators. Promoting preventive actions and raising public awareness are realistic ways for society to lessen cumulative exposure.

Finally, a multidisciplinary approach that spans laboratory science, clinical practice, public health policy, and international collaboration is necessary to address the reproductive hazards posed by EDCs. Protecting male reproductive health for present and future generations can be achieved by linking biological pathways to reproductive consequences and converting these understandings into preventive and regulatory measures.

## Figures and Tables

**Table 1:** Main biological pathways by which EDCs affect male reproduction.

Pathway/Mechanism	Example EDCs	Mode of Action	Consequences for Male Reproduction
Hormone receptor interaction	BPA, phthalates	Bind to ERα/Erβ Inhibit steroidogenic enzymes Disrupt AR/thyroid receptors	Altered gene expression Decreased testosterone Impaired spermatogenesis
HPG axis interference	Phthalates, pesticides	Disrupt GnRH, LH, and FSH signaling	Reduced Leydig/Sertoli function Low testosterone Poor sperm maturation
Epigenetic modifications	BPA, phthalates	DNA methylation Histone modification Altered ncRNA	Transgenerational reproductive effects Poor sperm quality
Oxidative stress and apoptosis	Multiple EDCs	ROS generation Mitochondrial dysfunction	Sperm DNA damage Apoptosis Infertility
Metabolic/Thyroid disruptions	PCB Organochlorines	After thyroid hormone signaling Receptor crosstalk	Impaired testicular development and energy metabolism

**Table 2:** Impact of EDCs on male fertility: Mechanistic vs. Epidemiological Evidence.

Aspect	Mechanistic Evidence	Epidemiological/Clinical Evidence
Sperm quality	BPA → mitochondrial dysfunction; oxidative DNA damage (animal)	BPA and phthalates associated with reduced sperm motility, concentration, and increased DNA fragmentation in men [31,32,33,34]
Hormonal imbalance	AR antagonism, steroidogenesis inhibition (cell/animal)	Phthalate metabolites and BPA biomarkers correlated with lower serum testosterone and altered LH/FSH ratios in population studies [30,35,36,37]
Testicular morphology	Seminiferous tubule degeneration, vacuolization of Sertoli cells, reduced Leydig cell counts (rats, mice)	Limited biopsy evidence; indirect clinical associations with infertility and reduced sperm counts [15,38]
Epigenetics	BPA and phthalates alter sperm DNA methylation, histone retention, and ncRNA expression (transgenerational animal models)	Human studies show altered sperm DNA methylation and microRNA profiles linked to urinary EDC biomarkers

**Table 3:** Summary of risk assessment and regulatory perspectives on EDCs.

Aspect	Description	Examples/Key Points
Exposure Routes	EDCs enter the body via ingestion, inhalation, and dermal absorption.	- Food and water (BPA, pesticide residues) - Inhalation of volatile compounds (flame retardants, solvents, occupational exposure) - Dermal absorption (phthalates, parabens, cosmetics, plastics) - Cumulative low-dose and mixture effects amplify risks [4,42,43,44]
Biomonitoring and Risk Assessment	Biological samples are used to measure exposure and link it to outcomes. Risk assessment establishes safe thresholds.	- Matrices: urine, blood, semen, and tissue - Reference values: ADI (acceptable daily intake) and NOAEL (no-observed-adverse-effect level) - Challenges: low-dose non-monotonic responses, mixture effects not fully captured [45,46,47]
Regulatory Frameworks	Multiple agencies provide guidelines, but critical gaps persist.	- EU: EFSA guidelines, REACH regulation - US: FDA & EPA regulation of consumer products, pesticides, and food-contact materials - Global: UNEP and WHO environmental monitoring and public health guidance - Gaps: insufficient testing of endocrine activity, lack of consideration for mixtures, sensitive developmental windows, and chronic low-dose effects [48,49,50,51]
Challenges and Future Directions	Improved tools and frameworks are needed for effective regulation.	- Integration of toxicological, epidemiological, and mechanistic evidence - Advanced tools: computational modeling, omics, high-throughput in vitro screening - Priorities: address low-dose and transgenerational impacts, reduce exposure during critical developmental periods, strengthen public health strategies [52,53,54,55]

## Data Availability

No new data were created or analyzed in this study.
